# Effects of reduced milking frequency before dry-off on systemic inflammation, subsequent performance, and health outcomes in dairy cows

**DOI:** 10.3168/jdsc.2026-1088

**Published:** 2026-06-25

**Authors:** J. Gao, C.G. Savegnago, T.N. Marins, A.M. Roper, N.L.P. Kant, J. Bohlen, S. Tao

**Affiliations:** Department of Animal and Dairy Science, University of Georgia, Athens, GA 30602

## Abstract

This study evaluated the effects of reduced milking frequency before dry-off on systemic inflammation, stress, and subsequent lactation performance and health outcome in primiparous (PRIM) and multiparous (MULT) Holstein dairy cows in a commercial dairy farm. On d −12 relative to dry-off (RTD), late-lactation cows were assigned to 1 of the 2 treatments according to the even or odd number of the last digit of ear tag: (1) reduced milking frequency (RMF; cows were milked 2×/d for 12 d before dry-off by omitting the last milking of the day; n = 516); or (2) control (CON; cows were milked 3×/d; n = 480). Cows in both treatments had similar parity, days carried calf, and milk yield at enrollment. At dry-off, all cows received intramammary antibiotic therapy followed by internal teat sealant according to the farm protocol. Blood samples were collected from a subset of cows (n = 60/treatment) on d −14, +1 (the day of dry-off), +2, and +9 RTD to measure plasma haptoglobin and serum cortisol. Composite milk samples were collected from all cows within 3 d after calving for on-farm bacteriological culture, but no milk culture was performed before dry-off. Milk yield, health events, and herd removal were monitored through 280 DIM in the subsequent lactation. Reduced milking frequency decreased daily milk yield by ∼48% in both PRIM cows (15 vs. 29 kg/d) and MULT cows (16 vs. 31 kg/d) on the first day of the treatment. This difference gradually narrowed as cows approached dry-off, reaching 7.7% in PRIM cows (24 vs. 26 kg/d) and 14.2% in MULT cows on the day of dry-off (24 vs. 28 kg/d). Reduced milking frequency did not affect plasma haptoglobin or serum cortisol in either parity around dry-off. At calving, RMF increased the incidence of positive milk cultures in MULT cows (13% vs. 7%), primarily driven by environmental bacteria (11% vs. 5%), but had no effect on incidence of positive milk culture in PRIM cows. Reduced milking frequency had no effects on performance or postpartum diseases incidences in the subsequent lactation but tended to increase the risk of herd removal in PRIM cows (19.9% vs. 13.7%). In conclusion, RMF before dry-off decreased milk yield but did not alter systemic inflammatory or stress responses around dry-off or improve subsequent lactation performance or postpartum health outcomes. Moreover, RMF may adversely affect intramammary infection during the dry period in MULT cows and herd retention in PRIM cows.

## Graphical Abstract

**Figure:**
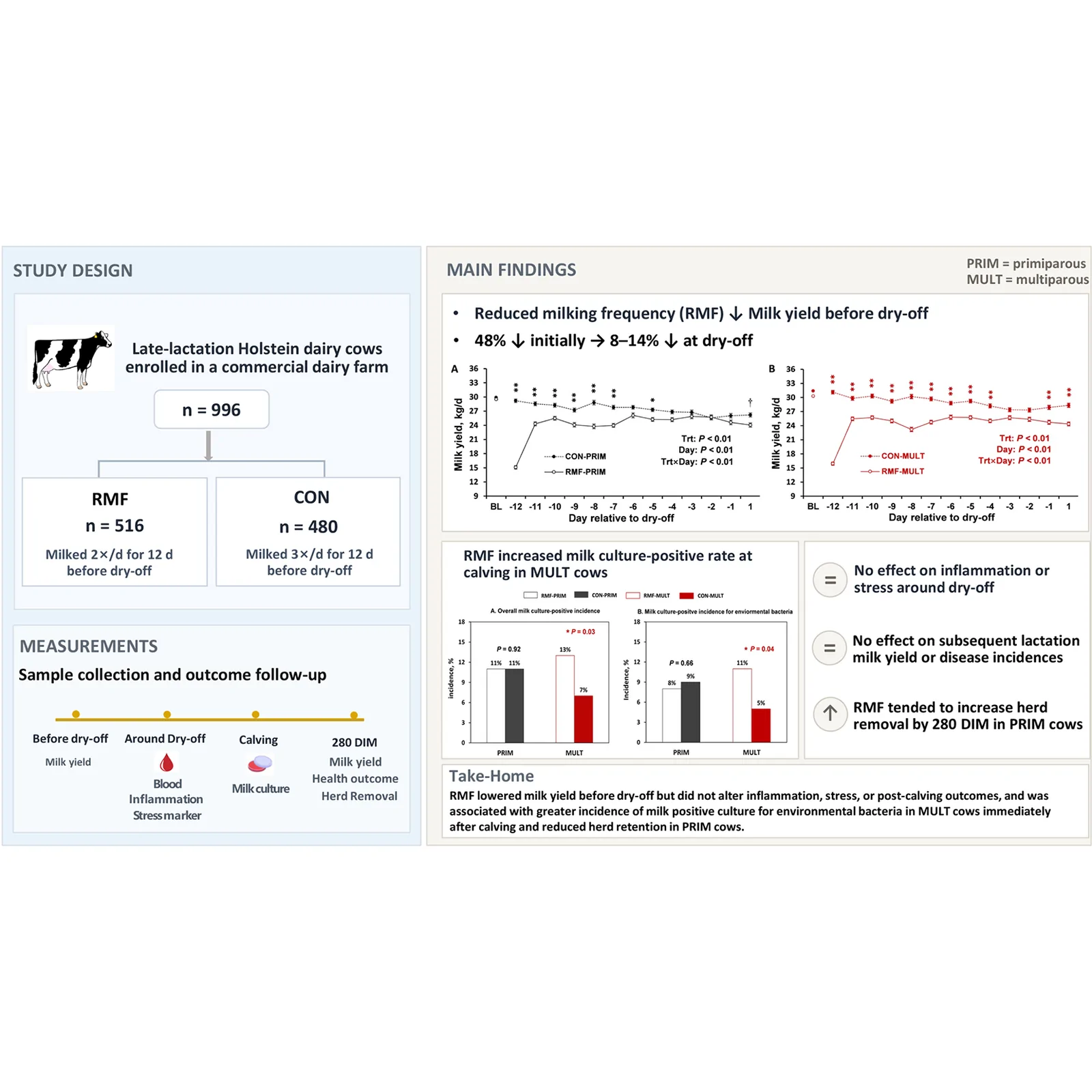

Transition from lactation to the dry period is challenging for dairy cows. Previous studies have reported marked increases in biomarkers related to stress and inflammation following dry-off (Putman et al., 2018; Gao et al., 2025), indicating upregulated stress and inflammatory responses. The magnitude of these systemic responses seems to be associated with milk yield at dry-off. Mezzetti et al. (2020) reported that cows with high milk yield (>15 kg/d) had a more pronounced inflammatory response, as indicated by the greater plasma haptoglobin concentration after dry-off. Similarly, Bertulat et al. (2013) found that fecal glucocorticoid metabolites increased after dry-off in cows across different production levels, but the increase in cows with higher milk yield had greater magnitude and was sustained longer, indicating a stronger stress response. Collectively, these studies suggest that higher milk yield at dry-off exacerbates systemic inflammatory and stress responses in the early dry period. Additionally, greater milk yield at dry-off increases the risk of intramammary infection during the dry period and early lactation. Rajala-Schultz et al. (2005) reported that, when cows produce above 12.5 kg/d at dry-off, every 5-kg increase in milk yield increased the risk to develop environmental intramammary infection by 77%. Similarly, Newman et al. (2010) reported that cows producing above 115 kg of milk during the last week of lactation were 7.1 times more likely to have infected mammary quarters after parturition than those producing 75 kg or less in the last week of lactation.

Accordingly, reducing milking yield before dry-off could minimize systemic inflammatory and stress responses and the risk of intramammary infection following the cessation of milking (Vilar and Rajala-Schultz, 2020; Cattaneo et al., 2023). Reduced milking frequency before dry-off, also referred to as gradual cessation of milking, is a management strategy to lower the milk yield before dry-off (Zobel et al., 2013; Larsen et al., 2021). However, to our knowledge, it remains unclear whether this practice attenuates systemic inflammation and stress response around dry-off, and whether these effects could affect the health and productivity of cows in the subsequent lactation. Therefore, the objective of this study is to evaluate the effects of reduced milking frequency in late lactation on systemic inflammation and stress response around dry-off and subsequent lactation performance and health outcomes. We hypothesized that gradual cessation of milking will reduce inflammatory and stress responses of cows following dry-off and improve milk production and cow health in the following lactation.

The experimental procedures and animal handling were approved by the University of Georgia Institutional Animal Care and Use Committee before study initiation (AUP no. A2020 08-001-A4). The experiment was conducted from February to April in a commercial dairy farm in southwest Georgia, USA. The farm housed ∼12,000 lactating Holstein dairy cows with an average daily milk yield of 47 kg/cow. During late lactation, cows were housed in freestall barns equipped with fans and soakers, fed the same lactating cow diet, and milked 3 times daily. All cows scheduled for dry-off from February to March were enrolled on d −21 relative to dry-off (**RTD**). On the day of enrollment, cows received routine vaccination, including Ultrabac 7/Somubac, (Zoetis Inc., Parsippany, NJ), J-Vac (Boehringer Ingelheim Inc., Duluth, GA), and Bovillis Guardian (Merck Animal Health Inc., Rahway, NJ). Vaccination was performed before dry-off to avoid interference from vaccination-induced systemic inflammation around dry-off. On d −12 RTD, cows were assigned to 1 of the 2 treatments according to the last digit of the ear tag. (1) In the reduced milking frequency (**RMF**) group, cows were milked 2 times daily before dry-off by omitting the last milking of the day (∼0930 and 1730 h; 16-h overnight interval; n = 516; ear tag ending with an odd number, 1, 3, 5, 7, or 9; RMF-primiparous cows [**PRIM**, first lactation before dry-off] = 218, RMF-multiparous cows [**MULT**, second or greater lactation before dry-off] = 298). (2) In the control (**CON**) group, cows were milked 3 times per day before dry-off (∼0900, 1700, and 0100 h; 8-h interval; n = 480; ear tag ending with even number: 0, 2, 4, 6, or 8; CON-PRIM = 213, CON-MULT = 267). This pseudo-randomization approach was adopted to facilitate animal identification and treatment administration by farm staff (Thompson et al., 2010; Gao et al., 2025). Cows assigned to the RMF group were moved from their original pen to another pen within the same freestall barn on d −12 RTD to allow omission of the last milking, whereas CON cows remained in their original pen. Cows in both treatments had similar parity (RMF: 2.0 ± 0.1; CON: 2.0 ± 0.1), days carry calves (RMF: 197.4 ± 0.1 d; CON: 197.5 ± 0.1 d), and average milk yield (RMF: 29.2 ± 0.3 kg/d; CON: 29.2 ± 0.4 kg/d) at enrollment. Values are presented as mean ± SD, and no differences were detected between treatments for these variables (*P* ≥ 0.32).

Cows at 216 to 221 d of gestation were dried off once weekly, and the day of dry-off was designated as d +1 RTD. After the last milking, all cows received blanket dry cow therapy, including intramammary infusion of an antibiotic (Spectramast DC, Zoetis Inc.) followed by an internal teat sealant (Orbeseal, Zoetis Inc.). Cows were then relocated to a freestall barn, fed a far-off dry cow diet and managed similarly thereafter. On 255 d of gestation, cows are moved to a close-up pen and vaccinated against *Escherichia coli* (J-Vac, Boehringer Ingelheim). After calving, cows were housed in lactating pens, milked 3 times a day, and fed the same lactating cow diet.

During the treatment period, 2 RMF cows developed mastitis and were moved to hospital pen before completing the treatment; 1 RMF cow died due to injury; and 3 RMF cows were sold because of lameness. Therefore, a total of 990 cows completed treatment and entered the dry period. During the dry period, 3 RMF cows died because of injury; 1 RMF cow and 2 CON cows died due to unidentified causes; 1 CON cows was sold after being diagnosed as open; and 2 CON cows were sold because of lameness. Consequently, a total of 981 cows, including 506 RMF cows (216 RMF-PRIM and 290 RMF-MULT) and 475 CON cows (211 CON-PRIM cows and 264 CON-MULT cows), entered the subsequent lactation.

Within 3 d after calving, aseptic composite milk samples were collected from all functional quarters of each cow for milk culture according to the established protocol of the farm. However, no milk culture was performed before dry-off. The on-farm culture system was based on triple-plate chromogenic medium. Culture outcomes were recorded in the herd management software (Dairycomp 305, Valley Agricultural Software, Madison, WI) as *Staphylococcus aureus* (**STA**), *Streptococcus* spp. (**STS**), *Streptococcus dysgalactiae* (**STPDY**), *Enterococcus* spp. (**ENTC**), *Enterobacter* spp. (**ENTB**), or no growth. For the subsequent analysis, STA was classified as contagious bacteria, whereas STS, STPDY, ENTC, and ENTB were classified as environmental bacteria.

Milk yield before dry-off and after calving through 280 DIM was recorded at each milking (Alpro, DeLaval, Kansas City, MO), and health events from calving to 280 DIM were recorded by farm staff. Data were extracted from farm management software. Health disorders were diagnosed and recorded by trained farm personnel or the herd veterinarian according to the farm protocol. Recorded health disorders included milk fever, retained placenta, metritis, ketosis, displaced abomasum, clinical mastitis, lameness, and removal from herd, including death or sale. Clinical mastitis cases were diagnosed by trained farm personnel during milking based on visible abnormalities in milk (e.g., flakes) observed during fore-stripping before milking.

Blood samples from a randomly selected subset of cows (n = 60 per treatment; 25 PRIM and 35 MULT cows within each treatment) were collected on d −14, +1, +2, and +9 RTD (1000 h). Samples on d +1 RTD were collected following the last milking, between 0900 and 1000 h. Blood was collected from coccygeal vessels into Vacutainer tubes (sodium-heparin and without anticoagulant, Becton Dickinson, Franklin Lakes, NJ). Tubes containing sodium-heparin were immediately placed on ice, and tubes without anticoagulant were left at room temperature to allow clotting. Within 3 h after collection, tubes were centrifuged at 1,700 × *g* for 30 min at 4°C to separate plasma and serum. Plasma concentration of haptoglobin was measured by a colorimetric assay reported by Cooke and Arthington (2013). Serum cortisol concentration was measured using a Cortisol ELISA kit (Arbor Assay, Ann Arbor, MI). Within each plate, the number of cows for each treatment was balanced and placed in alternative orders. The intra- and interassay CV were 1.3% and 8.1%, respectively, for haptoglobin, and 4.2% and 8.2%, respectively, for cortisol.

All statistical analyses were performed using SAS 9.4 (SAS Institute Inc., Cary, NC). Descriptive variables at enrollment, including parity, weekly milk yield, and days of gestation, were calculated using the UNIVARIATE procedure. Data from PRIM and MULT cows were analyzed separately. Repeated-measures data including daily milk yield before dry-off, weekly milk yield after calving, and circulating concentrations of haptoglobin and cortisol were analyzed using PROC MIXED. The model included fixed effects of treatments (RMF vs. CON), day (or week), and treatment by day (or week) interaction, with cows nested within treatment as the random effect. For milk yield before dry-off, average milk yield from d −17 to −13 RTD was included as a covariate. For circulating haptoglobin and cortisol, data collected on d −14 RTD were used as a covariate. The LSM ± SEM were reported. The incidence of positive milk culture after calving, and incidences of postpartum health events and herd removal from calving through 280 DIM, were computed using PROC FREQ. Binary variables were analyzd by logistic regression using PROC LOGISTIC. The model included treatment (CON vs. RMF), and the odds ratio and 95% CI were calculated and reported. Significance was defined as *P* < 0.05, and tendency was considered when 0.05 ≤ *P* < 0.10.

No treatment, day, or treatment by day effects were observed for plasma haptoglobin concentration in either PRIM or MULT cows from d +1 to +9 RTD (*P* ≥ 0.22; Supplemental Figure S1A and S1B, see Notes). Mean plasma haptoglobin concentrations were 133 and 135 μg/mL for CON-PRIM and RMF-PRIM cows, respectively, and 124 and 128 μg/mL for CON-MULT and RMF-MULT cows, respectively. Similarly, no treatment effect or treatment by day interaction was observed for serum cortisol concentration for either PRIM or MULT cows (*P* ≥ 0.28; Supplemental Figure S1C and D). However, day relative to dry-off affected serum cortisol concentration in both PRIM and MULT cows (*P* < 0.01). Regardless of treatment, serum cortisol concentration in PRIM cows was lower on d +9 RTD than d +1 and +2 RTD (8 vs. 17 and 16 ng/mL; *P* < 0.01). In MULT cows, serum cortisol concentrations were lower on d +2 and +9 RTD compared with d +1 RTD (11 and 8 vs. 16 ng/mL; *P* ≤ 0.02).

Treatment by day interactions (*P* < 0.01, Figure 1) were detected for daily milk yield before dry-off in both PRIM and MULT cows. On the first day of treatment (d −12 RTD), RMF decreased daily milk yield by ∼48%, from 29 to 15 kg/d in PRIM cows and from 31 to 16 kg/d in MULT cows. Thereafter, daily milk yield of RMF-PRIM cows was lower or tended to be lower from d −12 to −7, and on d −5 and +1 RTD compared with CON-PRIM cows (*P* ≤ 0.09, Figure 1A). Similarly, RMF-MULT cows had lower daily milk yield from d −12 to −4, and on d −2 and +1 RTD than CON-MULT cows (*P* < 0.01, Figure 1B).

**Figure 1:**
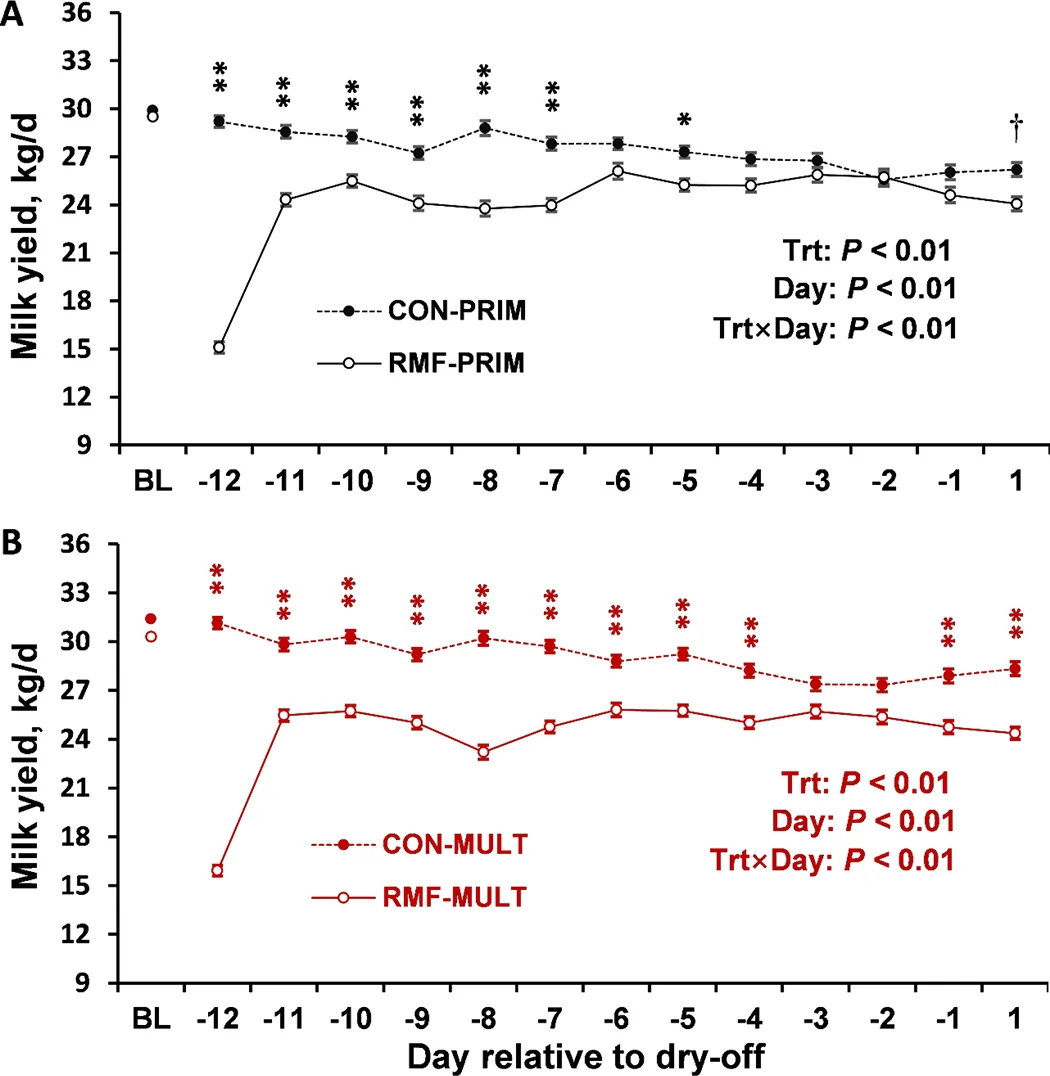
(A) Milk yield from d −12 to +1 relative to dry-off in primiparous cows assigned to reduced milking frequency (RMF-PRIM; milked 2×/d, n = 218) or control (CON-PRIM; milked 3×/d, n = 213). (B) Milk yield from d −12 to +1 relative to dry-off in multiparous cows assigned to reduced milking frequency (RMF-MULT, n = 298) or control (CON-MULT, n = 267). BL = average milk yield from d −17 to −13 relative to dry-off. **P < 0.01; *0.01 ≤ *P* < 0.05. †0.05 ≤ *P* < 0.10. Error bars represent SEM. Trt = treatment.

Among 926 milk culture results recorded, 96 samples (10.37%) were culture positive, including 60.42% (58/96) identified as STS, 18.75% (18/96) as STA, 15.63% (15/96) as ENTC, 3.12% (3/96) as STPDY, and 2.08% (2/96) as ENTB. No treatment effects were detected for overall culture-positive incidence or culture-positive incidence for environmental bacteria in PRIM cows (*P* ≥ 0.66, Figure 2). In contrast, in MULT cows, RMF increased overall culture-positive incidence (13% vs. 7%) and culture-positive incidence for environmental bacteria (11% vs. 5%) compared with CON cows (*P* ≤ 0.04). No differences were observed for culture-positive incidence for contagious bacteria in either parity (*P* ≥ 0.55, data not shown).

**Figure 2:**
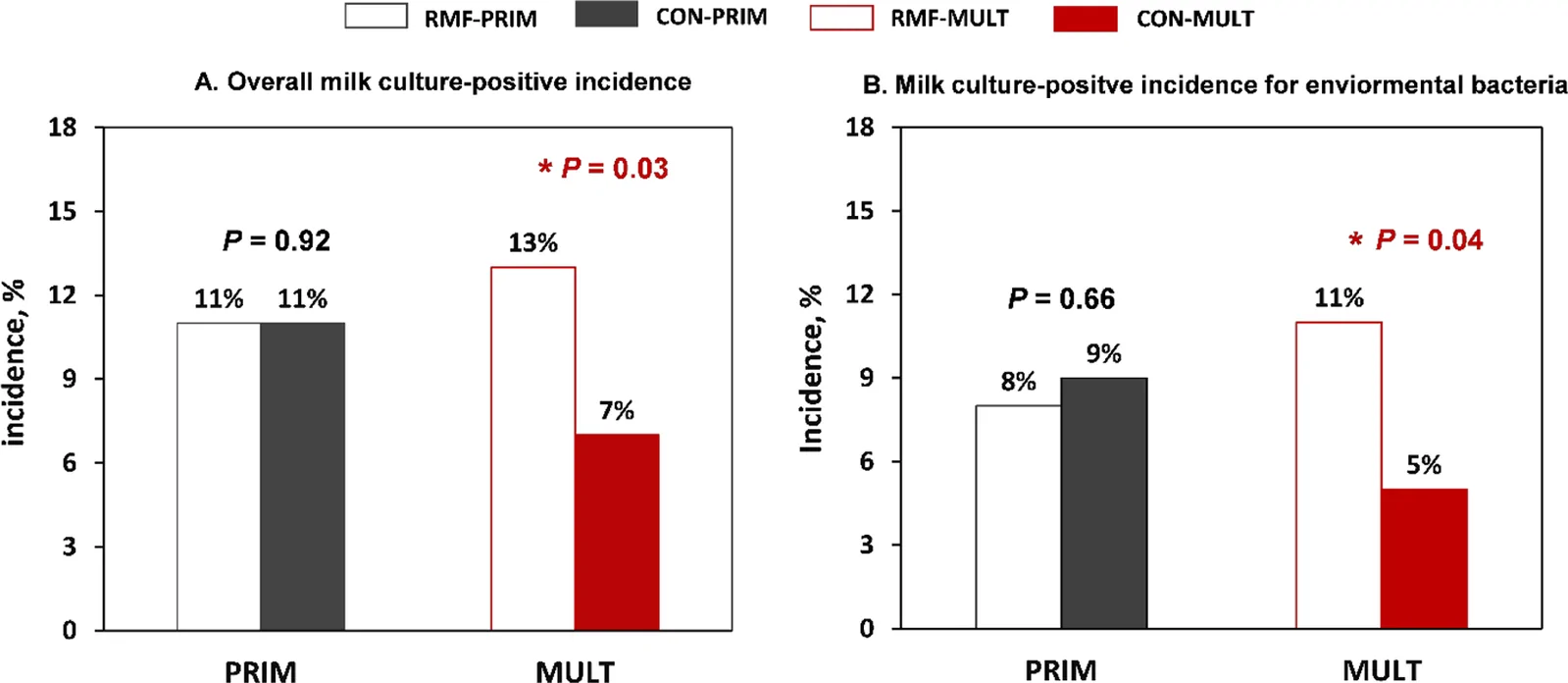
(A) Overall culture-positive incidence and (B) culture-positive incidence for environmental bacteria (including *Streptococcus* spp., *Streptococcus dysgalactiae*, *Enterococcus* spp., and *Enterobacter* spp.) in milk samples collected within 3 d after calving from primiparous (PRIM) and multiparous (MULT) cows assigned to reduced milking frequency (RMF; milked 2×/d) or control (CON; milked 3×/d) for 12 d before dry-off. RMF-PRIM, n = 216; CON-PRIM, n = 211; RMF-MULT, n = 290; CON-MULT, n = 264. *0.01 ≤ *P* < 0.05.

No treatment effects were detected for weekly milk yield in the subsequent lactation in either PRIM or MULT cows (*P* ≥ 0.35, Table 1). Similarly, treatment did not affect incidences of milk fever, retained placenta, metritis, ketosis, displaced abomasum, clinical mastitis, or lameness from calving through 280 DIM in either parity (*P* ≥ 0.13). In PRIM cows, RMF tended to increase herd removal in the subsequent lactation compared with CON (19.9% vs. 13.7%; *P* = 0.09). No treatment difference in herd removal was observed in MULT cows (*P* = 0.29).

**Table 1:** Subsequent lactation milk yield, health outcomes, and herd removal from calving through 280 DIM in primiparous and multiparous cows assigned to reduced milking frequency (RMF; milked 2×/d) or control (CON; milked 3×/d) for 12 d before dry-off

Item	Treatment		Estimate^1^	*P*-value
	CON	RMF		
Primiparous cows				
Weekly milk yield, kg/d	44.95	44.89	0.44	0.94
Milk fever,^2^ %	0 (0/211)	0 (0/216)	—	—
Retained placenta, %	3.32 (7/211)	6.48 (14/216)	2.02 (0.79–5.10)	0.13
Metritis, %	6.64 (14/211)	7.87 (17/216)	1.20 (0.58–2.50)	0.62
Ketosis, %	5.69 (12/211)	4.63 (10/216)	0.81 (0.34–1.91)	0.62
Displaced abomasum, %	0.47 (1/211)	0.46 (1/216)	0.98 (0.06–15.71)	0.98
Clinical mastitis, %	22.75 (48/211)	26.85 (58/216)	1.25 (0.80–1.94)	0.33
Lameness, %	9.00 (19/211)	12.50 (27/216)	1.44 (0.78–2.68)	0.25
Herd removal, %	13.74 (29/211)	19.91 (43/216)	1.56 (0.93–2.61)	0.09
Multiparous cows				
Weekly milk yield, kg/d	47.13	46.59	0.41	0.35
Milk fever, %	1.89 (5/264)	2.41 (7/290)	1.28 (0.40–4.08)	0.68
Retained placenta, %	6.06 (16/264)	6.90 (20/290)	1.14 (0.58–2.26)	0.69
Metritis, %	6.44 (17/264)	7.59 (22/290)	1.19 (0.62–2.30)	0.60
Ketosis, %	12.50 (33/264)	15.52 (45/290)	1.29 (0.79–2.09)	0.31
Displaced abomasum, %	1.89 (5/264)	2.76 (8/290)	1.47 (0.48–4.55)	0.50
Clinical mastitis, %	32.20 (85/264)	36.90 (107/290)	1.23 (0.87–1.75)	0.25
Lameness, %	18.56 (49/264)	20.00 (58/290)	1.10 (0.72–1.68)	0.67
Herd removal, %	28.79 (76/264)	24.83 (72/290)	0.82 (0.56–1.19)	0.29

1 Estimate = SEM for average daily milk yield and odds ratio with 95% CI for health-event outcomes.

2 No statistical analysis was performed.

Reduced milking frequency is a strategy to decrease milk yield before dry-off to facilitate the transition into the dry period. Previous studies have reported that milk yield reduction by RMF ranges from 13% to 33%, depending on both the magnitude and the duration of the reduction in milking frequency (Tucker et al., 2009; Gott et al., 2016). For instance, Zobel et al. (2013) evaluated a 5-d gradual cessation protocol consisting of a series of single- and double-skipped milkings from twice-daily milking before dry-off and reported a 22% reduction in milk yield at final milking compared with abrupt cessation. In the present study, the RMF period was extended to 12 d before dry-off, allowing evaluation of the milk yield response over a prolonged period. We observed an ∼48% reduction in milk yield by RMF in both PRIM and MULT cows on the first day of treatment. In this study, to achieve RMF, cows were relocated to a different pen under the same barn. von Keyserlingk et al. (2008) reported an ∼8% decrease in milk yield on the first day of regrouping, which is likely associated with acute behavioral disruptions. Thus, the initial reduction in milk yield observed in RMF cows may reflect the combined effects of reduced milking frequency, stress responses, and social disruption due to regrouping. When approaching dry-off, the milk yield difference between RMF and CON cows gradually narrowed, reaching ∼8% in PRIM cows and 14% in MULT cows at dry-off. This pattern suggests partial rebound in milk yield during the intervention period. It is important to recognize that the effectiveness of gradual milking cessation should be interpreted in the context of the RMF strategy used in specific studies. In the current study, milking frequency was reduced from 3 to 2×/d, whereas previous studies reduced milking frequency from 3 or 2 to 1×/d before dry-off (Rajala-Schultz et al., 2018; Larsen et al., 2021). This distinction is important because reducing milking frequency from 3 to 2×/d resulted in an ∼16-h overnight milking interval, whereas reducing milking frequency to 1×/d results in an ∼24-h milking interval. Therefore, the magnitude of milk yield reduction, milk accumulation, udder pressure, and risk of milk leakage may differ among RMF strategies. In addition, the 12-d RMF period used in the current study allowed us to evaluate milk yield responses over a longer intervention period. Consistent with this observation, France et al. (2022) reported an 8.8% overall reduction in milk yield when milking frequency was reduced from 2.7 to 1.6 milkings daily during the final 2 wk of lactation in an automatic milking system.

Although RMF reduced milk yield before dry-off, the circulating haptoglobin did not differ between PRIM and MULT cows. In this study, RMF cows produced ∼24 kg/d before dry-off, which remained well above the 15 kg/d threshold previously reported to differentiate the inflammatory responses following dry-off between low- and high-producing cows (Vilar and Rajala-Schultz, 2020). These findings suggest that the moderate decrease in milk yield (8%–14%) by RMF in the last 12 d of lactation in high-producing cows was insufficient to alter systemic inflammation around dry-off. However, it is also possible that the infrequent blood sampling schedule in the present study prevented us from detecting different inflammatory responses following dry-off. Additionally, we have previously reported that high-producing cows (26 kg/d at dry-off; Gao et al., 2026) did not exhibit significant upregulation in systemic inflammation following milking cessation. This is consistent with the present study and also explains the lack of differences in inflammatory responses between RMF and CON cows. Previous studies have reported that the circulating cortisol concentration increased on or after the day of dry-off, indicating an upregulated stress response (Putman et al., 2018; Gao et al., 2025, 2026). In contrast, circulating cortisol remained the same or decreased following dry-off in the current study. The discrepancy between studies may reflect differences in dry-off management, including but not limited to feeding regimens before dry-off (feed restriction vs. ad libitum), housing options for dry cow facility (pasture vs. freestall barn), and distance and means of transportation from lactating pen to dry cow facility.

The early dry period is a vulnerable period for mammary health in dairy cows. Cessation of milking is associated with udder engorgement and greater risk of milk leakage, which increases the susceptibility of intramammary infection, particularly the environmental pathogens during the dry period (Bradley and Green, 2004; Vilar and Rajala-Schultz, 2020). However, udder engorgement and associated milk leakage may occur not only after the complete cessation of milking but also during the time when milking frequency is reduced. Gott et al. (2016) reported that multiparous cows milked 1×/d during the final week of lactation experienced greater milk leakage before dry-off and had 2.78-fold greater odds of quarter-level intramammary infection at calving compared with cows milked 2×/d. These findings suggest that RMF may increase the risk of milk leakage before dry-off and subsequent intramammary infection, thereby negatively affecting mammary health. However, it is important to acknowledge that udder engorgement and milk leakage were not directly evaluated, and pre-dry-off milk samples and cow SCC data were unavailable in the current study. Therefore, although this mechanism is biologically plausible and supported by previous literature, its contribution to the greater incidence of positive milk cultures for environmental bacteria observed in RMF-MULT cows remains speculative and warrants further investigation. In this study, we also observed a parity-dependent response regarding the incidence of positive milk culture, such that the greater incidence of positive milk culture due to RMF was observed only in MULT cows but not in PRIM cows. This may be because primiparous cows possess a more effective anatomical and physiological barrier against milk leakage, including a more robust teat-canal seal and enhanced streak-canal integrity (Cousins et al., 1980; Dingwell et al., 2004). Indeed, Gott et al. (2016) reported that cows ending their third or greater lactation had 3.0-fold greater odds of milk leakage than cows ending their first or second lactation when milking frequency was reduced from 2 to 1×/d during the last week of lactation.

Despite the difference in postpartum milk culture results, clinical mastitis incidence during the next lactation did not differ between treatments in either PRIM or MULT cows. This may be due to the greater incidence of clinical mastitis (23%–37%) compared with the incidence of positive milk culture (8%–11%), which could mask potential differences in clinical cases originating from bacterial infection observed immediately after calving. These data also suggest that effective management around dry-off is critical for minimizing mammary infections during the dry period; however, this alone does not guarantee optimal mammary health at the herd level. Clinical mastitis incidences are also influenced by numerous management, nutritional, environmental, and cow-level factors during lactation beyond intramammary infections developed during the dry period. Treatment did not affect incidences of other postpartum diseases or milk yield in the subsequent lactation. These findings are consistent with those of Wieland et al. (2023), who reported that, compared with abrupt cessation of milking, gradual cessation of milking before dry-off did not affect clinical mastitis incidence, SCC, or milk yield in early lactation. However, RMF-PRIM cows tended to have a greater incidence of herd removal up to 280 DIM than CON-PRIM cows, suggesting that the stress of regrouping and RMF in late lactation may reduce the survival of PRIM cows.

In conclusion, reduced milking frequency for 12 d before dry-off moderately decreased milk yield without altering systemic inflammation or stress response around milking cessation, subsequent lactation performance, and health in the subsequent lactation. However, RMF was associated with a greater incidence of positive milk cultures at calving in MULT cows and reduced head retention in PRIM cows. A limitation of the current study was that the milk culture data before dry-off and milk SCC data in the subsequent lactation were not available. Therefore, future studies need to be conducted in multiple farms with different management styles, and more comprehensive mammary health measurements are still required to validate the results reported herein.
